# Preschoolers and the Endowment Effect

**DOI:** 10.1371/journal.pone.0109520

**Published:** 2014-10-09

**Authors:** Sergio Da Silva, Bruno Moreira, Newton Da Costa

**Affiliations:** 1 Graduate Program in Economics, Federal University of Santa Catarina, Florianopolis, Santa Catarina, Brazil; 2 Federal Institute of Minas Gerais, Formiga, Minas Gerais, Brazil; University of Leicester, United Kingdom

## Abstract

We show that preschoolers exhibit the endowment effect as evidenced by experiments where children generally chose to keep their own toys rather than trading them for similar ones. Furthermore, we relate the emergence of this effect to children's innate psychobiological traits—emotional state, gender, handedness, and digit ratio. The trials were conducted with 141 children across 6 kindergartens. We also found support that children, like adults, exhibit a preference for physical possession as opposed to ownership. As with adults, emotions also seem to matter, as children who were described as quiet and calm were more likely to present the endowment effect. Also of note, right-handed children described as quiet were more likely to exhibit the phenomenon. Furthermore, female children were generally found to be calmer than males, while males tended to be more fearful than females. This result was also previously found in teenagers.

## Introduction

Merely possessing an object raises its value to its owner. This “endowment effect” occurs robustly in both laboratory and natural settings, and its psychological and neural mechanisms are just now beginning to be understood. From the standpoint of standard economic theory, the effect is unexpected because when given a choice between two goods, rational individuals choose the good with greater value. However, the effect can still be explained using microeconomic analysis [1], in that a reluctance to trade due to the endowment effect can simply be regarded as a mistake. If it is a misunderstanding, then people trade too little, thereby foregoing the benefits of trade. An alternative explanation of the endowment effect is levied by cognitive psychology and prospect theory. Here, prospect theory cites that choice is greatly influenced by a combination of the theory's two main “ingredients:” 1) loss aversion, such that losses are weighed more heavily than equal gains and 2) taste variations in reference to a baseline. Which explanation is right? This cannot be decided on the basis of theory alone.

Neuroscience evidence favors prospect theory [2]. The endowment effect, in particular, occurs on the right side of the insule, which is associated with the prediction of loss [3]. The effect has more to do with fear of losing a desired possession than wanting it in the first place.

Loss aversion is built into the automatic evaluations of the human mind, and it does require slow thinking for rationality to take control. The human thinking and decision making are biological adaptations rather than engines of pure rationality. It should then come as no surprise that the endowment effect was also observed in nonhuman primates, thus suggesting deep evolutionary origins. In humans the effect occurs between goods that are held for use, but not for exchange. Likewise, in other primates the effect occurs in food, but not for tools. Of note, even some birds are found to behave in accordance with prospect theory [4].

A recent study, however, found that hunter-gatherers from modern-day Tanzania did not show the endowment effect [5] challenging the idea that humans displayed the effect in their evolutionary past. But the fact that these Hadza people did not exhibit the effect may be explained by the social pressures likely present in such a communal group. After all, the effect emerges because one simply values one's own private property more so in the absence of legal institutions which ensure third-party contract enforcement [6]. In this case communal pressures are seen as the equivalent of such legal institutions.

If the endowment effect is indeed a mistake, market experience should lessen its effects over time. Harbaugh, Krause and Vesterlund [7] tested this hypothesis by considering a participant pool with differing market experience that included children aged five to ten along with undergraduates. They found no evidence that the endowment effect decreases with age.

In this research, after demonstrating that preschoolers show the endowment effect (thus replicating [7]), we go further and relate the emergence of this effect to children's innate psychobiological traits. Previous research has shown that emotions interfere with the endowment effect in adults [8]. This research considers whether the emotional state of children also plays a role in the effect. Additionally, we also compile data on gender, handedness, and digit ratio. Handedness is a neurological characteristic [9]. Furthermore, the digit ratio is a proxy for fetal testosterone such that high fetal testosterone levels or low fetal estrogens are accompanied by low 2D:4D digit ratios [10].

## Materials and Methods

Our experiments were conducted from September to December 2012 at 6 kindergartens (5 high income and 1 lower income school) in the southern Brazilian city of Florianopolis. The research received approval from the Ethical Committee of the Federal University of Santa Catarina (approval number: 057/08; date: 10 April 2008; published: 28 April 2008). All the participating kindergartens, along with the children's parents, provided written consent.

**Figure 1:**
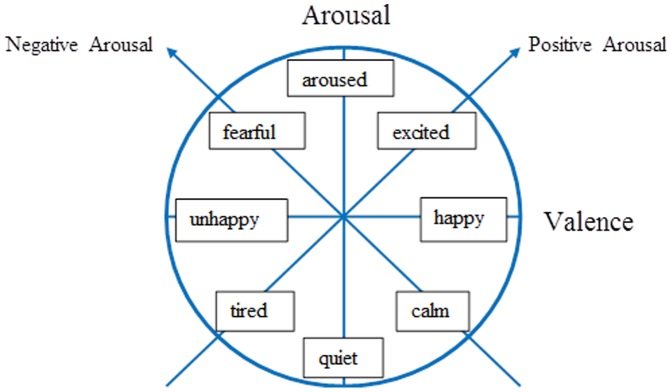
Graphical representation of the circumplex model of affect based on [11].

We collected data on 141 children (67 males and 74 females) aged four to six (data available at http://dx.doi.org/10.6084/m9.figshare.1160510). School teachers reported the emotional state of the children during the experiments in terms of the standard “affective circumplex” (Figure 1). The circumplex model of affect “was offered both as a way psychologists can represent the structure of affective experience, as assessed through self-report, and as a representation of the cognitive structure that laymen utilize in conceptualizing affect” [11]. Figure 1 was then presented to the teachers, who picked one state for each child and then reported their choice to the experimenter by the end of lunch time. They classified eight boys as happy, 12 as calm, 11 as quiet, and 36 as fearful. Nine girls were classified as happy, 29 as calm, 18 as quiet, and 18 as fearful. Female children were calmer than boys, and males were more fearful than females, a result that also appears in teenagers [12]. In our sample, 105 children were right-handed (62 females and 43 males) and 36 were left-handed (12 females and 24 males). As expected for adults and preschoolers [13], our sample also showed that the average 2D:4D ratio for males (mean  = 0.943; SD = 0.027) was lower than that of females (mean  = 0.981; SD = 0.014), and the *t* test for mean difference showed a *p*-value <0.001.

**Figure 2:**
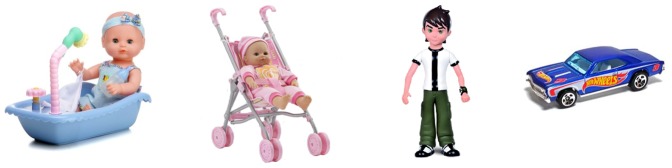
Pairs of goods presented to the little girls and boys.

Kindergarten teachers were first approached by the experimenter and asked about the favorite toys that their students brought to school on the days they were allowed. They reported that a baby bath tub and a baby carriage (as seen in Figure 2) for girls, and Hot Wheels miniature cars and Ben 10 toy dolls for boys were popular choices.

We then purchased twenty units of each toy shown in Figure 2 since no class had more than twenty children. We then gave a sample of each toy to the teachers to show to the parents on the days preceding the experiment. Parents were instructed by the teachers to have their children bring toys as similar as possible to those in Figure 2 on the day of the experiment. Most parents actually decided to purchase similar toys when no similar one was available at home. In 5 kindergartens of higher income, the vast majority of the parents actually purchased one of the toys described in Figure 2. In the lower income kindergarten approximately 20 percent of the parents did not purchase a toy. Despite that, the results in the lower income kindergarten did not differ from those of higher income ones suggesting that prior exposure to similar toys did not affect results. On the day of the experiment, children brought their own toys and also received a similar toy from the experimenter. Miniature cars or Ben 10 dolls were provided to the boys depending upon which toy the child brought from home. Similarly, baby bath tubs or baby carriages were presented to the girls. In all cases it was explained to the children that these toys were being lent, rather than given, to them. However, lending instead of giving makes no difference on the endowment effect [14]. Reb and Connolly [14] found a significant effect of possession, but not of factual ownership, on valuation of an object. This implies that the endowment effect does not rely on factual ownership, per se, but rather is the result of subjective feelings of ownership induced by the possession of an object. Boys and girls spontaneously played with same-sex peers during lunch time and played with their own toys as well with those borrowed from the experimenter. Also noteworthy, we observed that many children opted to share their toys. After playing with the toys roughly 25 minutes, the experimenter approached each kindergartner in private. The same experimenter (B.M.) conducted all the trials. The kids were then asked a series of questions, always in the same order, relating to their preferences between their own toy and the borrowed one:

1. Do you want to keep your toy or trade it for the one you borrowed from me?
2. Would you swap your favorite toy for this one borrowed from me?
3. Would you trade your future Christmas present for this toy borrowed from me?

For the endowment effect to occur, “yes” will be an answer to all of the three questions. Question 1 is expected to generate more affirmative answers than question 2; and likewise question 2 should receive more affirmatives than question 3. Question 1 addresses the possession of the borrowed toy. Question 2 involves a favorite toy that does not necessarily coincide with the one brought from home. Question 3 involves a hypothetical trade for a future Christmas present. For these reasons, trades were actually executed only for Question 1 in the cases where children accepted the deal. After the children answered these questions, the experimenter measured their digit ratios, assessed their handedness, and marked their gender. The 2D:4D ratio was measured by the experimenter after tracing the contour of each child's right hand on a sheet of white paper.

When granted a choice between two toys, an endowment effect exists if the probability that a child chooses good A is higher than good B when initially endowed with good A. For every good, the likelihood of choosing that good increases when the child was previously endowed with it. We then considered the model
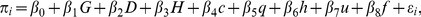
(1)where 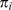 stands for the probability of endowment effect for the three questions 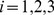; if the effect exists then 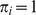. The 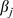 are coefficient vectors capturing the impact on 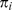 of an explanatory variable 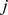. Variable 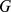 is a dummy for gender (female  = 0; male  = 1); 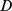 is digit ratio; and 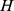 is a dummy for handedness (left-hander  = 0; right-hander  = 1). Variable 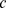 stands for calm; 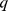, for quiet; 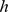, for happy; 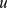, for unhappy; and 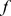, for fearful. Such dummies assume a value of zero for a given attribute and one for all the remaining others. 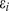 is an error term. The selection of an explanatory variable was made using stepwise, backward, and forward regressions.

## Results

For question 1, the endowment effect appeared in 110 children (78.01%); for question 2, 105 children (74.47%); and for question 3, 99 children (70.21%) (*p*-value <0.0001; chi-squared test for the three trials). Such results replicate [7] where five-year olds showed the effect between 65% and 75% of the trials. The results also confirm [14] as to the importance of physical possession, as opposed to ownership, because the effect is shown to be stronger in question 1.

A logit model selected the variable “quiet” as statistically significant (*p*-value <0.008; *z* = 2.64) for explaining the endowment effect in question 1:



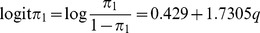
(2).

The variable “calm” was selected in question 2 (*p*-value <0.002; *z* = 3.06):



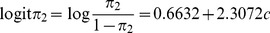
(3).

And the variables “quiet” (*p*-value <0.006; *z* = 2.75) and “right-hander” (*p*-value <0.0001; *z* = 4.58) were selected for question 3:



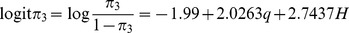
(4).

The results in equations (2) − (4) can be translated into probability terms. For question 1, children who were described as quiet were 90% more likely to show the endowment effect. For question 2, calm children were 95% more likely to display the effect and for question 3, right-handed children described as quiet were 94% more likely to exhibit the effect. Interestingly, handedness plays a role when expectations are involved. Quiet and calm are positive emotional states, and positive emotions entail positive projections onto goods, thereby inducing the desire to keep them. This may explain the relationship between the endowment effect and these emotional states. This result was also found in adults [15], [16]. We show these relationships occur in children as well.

## Conclusion

Preschoolers were presented three questions to elicit the endowment effect. Using a sample of children aged four to six, we found support for the findings of Harbaugh, Krause and Vesterlund [7] (who considered children aged five to ten), and also for the prominence of physical possession as opposed to ownership, as previously shown in adults [14].

In the first elicitation, we found emotions matter for the endowment effect, as children who were described as quiet were more likely to present the effect. Female children were calmer than males, and males were more fearful than females, a result previously found in teenagers. Calm children were also more prone to the effect in the second elicitation. And right-handed children described as quiet were more likely to exhibit the effect in the third one. The role of positive emotions in the endowment effect was shown previously in adults [15], [16], and here we find it holds for children as well.

## References

[1] Hanemann WM (1991) Willingness to pay and willingness to accept: How much can they differ? American Economic Review 81: 635–647.

[2] Trepel C, Fox CR, Poldrack RA (2005) Prospect theory on the brain? Toward a cognitive neuroscience of decision under risk. Cognitive Brain Research 23: 34–50. 15795132 10.1016/j.cogbrainres.2005.01.016

[3] Knutson B, Wimmer GE, Rick S, Hollon NG, Prelec D, et al (2008) Neural antecedents of the endowment effect. Neuron 58: 814–822. 18549791 10.1016/j.neuron.2008.05.018

[4] Marsh B, Kacelnik A (2002) Framing effects and risky decisions in starlings. PNAS 99: 3352–3355. 11867709 10.1073/pnas.042491999 PMC122522

[5] Apicella CL, Azevedo EM, Fowler JH, Christakis NA (2014) Evolutionary origins of the endowment effect: Evidence from hunter-gatherers. American Economic Review 104: 1793–1805.

[6] Gintis H (2007) The evolution of private property. Journal of Economic Behavior & Organization 64: 1–16.

[7] Harbaugh WT, Krause K, Vesterlund L (2001) Are adults better behaved than children? Age, experience, and the endowment effect. Economics Letters 70: 175–181.

[8] Zhang Y, Fishbach A (2005) The role of anticipated emotions in the endowment effect. Journal of Consumer Psychology 15: 316–324.

[9] Da Silva S, Baldo D, Matsushita R (2013) Biological correlates of the Allais paradox. Applied Economics 45: 555–568.

[10] Manning JT, Scutt D, Wilson J, Lewis-Jones DI (1998) The ratio of 2nd to 4th digit length: A predictor of sperm numbers and concentrations of testosterone, luteinizing hormone and oestrogen. Human Reproduction 13: 3000–3004. 9853845 10.1093/humrep/13.11.3000

[11] Russell JA (1980) A circumplex model of affect. Journal of Personality and Social Psychology 39: 1161–1178. 10.1037//0022-3514.79.2.286 10948981

[12] Deb S, Chatterjee P, Walsh K (2010) Anxiety among high school students in India: Comparisons across gender, school type, social strata and perceptions of quality time with parents. Australian Journal of Educational & Developmental Psychology 10: 18–31.

[13] Moreira B, Matsushita R, Da Silva S (2010) Risk-seeking behavior of preschool children in a gambling task. Journal of Economic Psychology 31: 794–801.

[14] Reb J, Connolly T (2007) Possession, feelings of ownership and the endowment effect. Judgment and Decision Making 2: 107–114.

[15] Lin CH, Chuang SC, Kao DT, Kung CY (2006) The role of emotions in the endowment effect. Journal of Economic Psychology 27: 589–597.

[16] Martinez LF, Zeelenberg M, Rijsman JB (2011) Regret, disappointment and the endowment effect. Journal of Economic Psychology 32: 962–968.

